# Effects of Mentoring Speed Dating as an Innovative Matching Tool in Undergraduate Medical Education: A Mixed Methods Study

**DOI:** 10.1371/journal.pone.0147444

**Published:** 2016-02-09

**Authors:** Jennifer Guse, Eva Schweigert, Gerhild Kulms, Ines Heinen, Claudia Martens, Andreas H. Guse

**Affiliations:** 1 Department of Medical Psychology, University Medical Center Hamburg-Eppendorf, Hamburg, Germany; 2 Dean’s Office of Education and Students’ Affairs, University Medical Center Hamburg-Eppendorf, Hamburg, Germany; 3 Department of Biochemistry and Molecular Cell Biology, University Medical Center Hamburg-Eppendorf, Hamburg, Germany; Charité - Universitätsmedizin Berlin, GERMANY

## Abstract

**Objectives:**

Choosing the right mentor is crucial for effective mentorship. Yet, many medical students have difficulties finding a suitable mentor. Thus we developed mentoring speed dating (MSD) as a promising matching tool to connect students and faculty mentors successfully. The purpose of this study was to explore mentees’ and mentors’ experience with MSD and investigate the impact of MSD on the perceived mentorship quality and continuance of the mentoring relationship.

**Methods:**

The authors completed a mixed methods study at the University Medical Center Hamburg-Eppendorf, Germany, between June 2011 and March 2014. They conducted four focus groups with mentees and mentors who participated in a mentoring speed dating event and analyzed transcripts using conventional content analysis with inductive categorizing. In addition, three mentoring cohorts (two matched via MSD, one matched via conventional online profiles) were surveyed on mentorship satisfaction and the 1-year continuance of their mentorship was monitored. Fifteen mentees and fifteen mentors participated in the focus groups. The authors identified several themes such as short and long term benefits of MSD and fulfillment of expectations. Benefits included finding out about the personal connection, matching expectations, providing an efficient overview of candidates. The survey was completed by 93 students (n = 29 without MSD; n = 64 with MSD). Independent *t*-tests and multivariate analysis of variance were used to analyze the impact of MSD on student’s mentorship satisfaction.

**Results:**

There were significant differences in responses to the items “Commitment of mentor” (p = .019) and “Constructive feedback” (p = .038) among the students who attended MSD and the students without MSD. After one year far more mentoring relationships existed among those mentees who participated in MSD in comparison to the “no MSD group”.

**Conclusion:**

MSD is a valuable matching tool with beneficial effects on the mentorship quality. It enhances essential factors in the mentoring relationship such as commitment and satisfaction.

## Background

Due to the positive effects of mentoring on the professional development of medical students and young physicians, formal mentoring programs have gained popularity within academic medicine [1–4]. Mentoring is a complex phenomenon and numerous definitions exist in the literature. In the specific context of student-faculty mentoring we follow the approach of Meinel et al. [1]: Considering the definitions of Berk [5] and Buddeberg-Fischer [6] the authors specified three key elements and objectives of student-faculty mentoring relationships: (i) personal in nature, involving direct interaction; (ii) intended to be long-lasting; and (iii) characterized by an integrated approach including emotional and psychological support as well as direct assistance with career and professional development [1].

Several benefits of mentoring have been reported for mentees (students), mentors (faculty) and institutions (medical schools) [1, 3, 7, 8]. Nevertheless, mentoring relationships are time-consuming and challenging when facing multiple demands such as clinical, research and administrative duties [9, 10]. Thus, the successful matching of mentees and mentors is of great importance for formal mentoring programs to avoid ineffective mentoring experiences [11]. Formal mentoring does not seem to be as effective as the traditional, informal mentoring where the relationship occurs spontaneously and is based on a specific mutual trust [12–14]. However, the development of the mentee-mentor relationship and satisfaction with mentorship are essential aspects for the success of any mentoring process [15, 16].

Mismatches in terms of different values, attitudes or work styles are quite common in mentee-mentor relationships [11] and often lead to dysfunctionality in terms of less psychosocial and career support [17, 18]. Interestingly, the process of matching in formal mentoring programs is neglected in most empirical research [18–20].

In December 2011 we implemented an annual “mentoring speed dating” event to facilitate mentoring relationships between students and faculty mentors, who participated in a formal mentoring program that fosters early introduction to research and promotes academic careers [4]. Furthermore this mentoring program for medical students offers general advice, guidance and support provided by faculty mentors. Depending on the mentees’ actual interest for instance mentors give opportunities to visit a lab and colloquia and enable mentees to engage in research themselves, or mentors give advice to find a thesis theme and supervisor.

MSD, as an innovative matching tool, was adapted from the concept of “speed dating” [18, 21]. The concept of “speed dating” is based on the ‘thin-slices paradigm’ where people extract particular impressions in a dynamic process within 30 seconds to 5 minutes and thus, are able to give an intuitive judgment [22, 23].

The purpose of this study was to gain an in-depth understanding of MSD as an innovative matching tool to connect mentees (students) and faculty mentors. In particular, we were interested in both mentees’ and mentors’ view on and experience with MSD regarding the process of matching and the outcome in terms of satisfaction with the mentoring relationship. Furthermore we aimed to investigate effects of MSD by comparing the mentorship quality and the 1-year continuance of the mentoring of three mentoring cohorts, who were matched either via MSD or chose their mentors via online profiles.

## Methods

### Setting

The study was conducted at the University Medical Center Hamburg-Eppendorf (UKE), Germany between December 2010 and January 2014. We used a mixed methods approach including method- and data triangulation. Both qualitative (focus-groups) and quantitative data (cross sectional survey) were collected.

### Participants and MSD design

Second- and third-year medical students at the UKE who participated in the mentoring program for excellent students [4] since December 2010 (mentoring cohort 2010, n = 37), December 2011 (mentoring cohort 2011, n = 37) and November 2012 (mentoring cohort 2012, n = 34) were invited to participate in this study. 10% of second-year medical students at the UKE were invited on the basis of excellent academic merit and high interest in research to participate in the mentoring program for excellent students. The mentoring cohort 2010 chose their faculty mentors by viewing their online profiles only. The mentor profiles included information regarding the mentor’s specialization, career-related aspects, recreational interests, and the mentor’s expectations regarding mentorship. Mentor profiles were grouped by the mentors’ affiliation to one of five established research centers at the UKE. In contrast, the mentoring cohort 2011 and 2012 met their mentors via an innovative method called MSD. The approach is based on speed dating and conceptually addresses some of the matching issues of formal mentoring programs. The matching process was carried out in two stages. First, students were asked to complete an application form stating two preferred main areas of research, with the option to choose from two of five established research centers at the UKE. Each research center was portrayed briefly on internet including online profiles of associated mentors. Furthermore the application included a self-assessment of their current interest in research on a 6-point Likert scale. Finally, two open-ended questions asking for the goals students would like to achieve and students’ expectations of mentorship were added. Second, students were invited to an inaugural event of the mentoring program for excellent students. There they met all mentors associated with their preferred area of research during a MSD session. The inaugural event took place on a weekday evening in December 2011 and November 2012 at UKE and lasted from 5pm till approximately 8.30pm. At the beginning the five established research centers at the UKE were presented briefly, followed by an introduction of the MSD procedure. The MSD event was based on Cook et al.’s work in 2010 [21]. Students spent 5 minutes with each mentor. Both had the chance for specific questions relevant to mentorship and their ideas of research. Students were able to prepare their questions based on each mentors’ online profile. Respectively, mentors received the application of each student who was going to see him, prior to the speed mentoring event. Between the encounters participants had 5 minutes to take notes. All mentors were seated on the same floor in separate rooms. Rotation schedules were attached to each door. All students received a pseudonym, which was listed on these schedules. After talking to all mentors of one area of research students left a note with their preferred mentor, second and third choice for the organizers (JG, ES) of the event. Likewise mentors left a note with the names of their preferred candidates available in rank order or forwarded their choice by email to the organizer within three days after the inaugural event. In case matching was not successful, the organizers of the program recommended mentors with mentorship vacancy and the remaining students made individual appointments until they found a mentor they felt comfortable with. Likewise the mentor had to agree on the mentorship with the mentee, too, before the individual mentoring-relationship started. Information about the mentors’ cohorts are presented in Table 1. Not all available mentors have been chosen by mentees. Thus the table includes only mentors with at least one mentee. Mentors provided different capacity for mentees. The maximum number of mentees per mentor ranged from 1 to 4 mentees Due to the actual choice of mentees the mean number of mentees per mentor varied from M = 2.31 (SD = 1.14) to 1.76 (0.89) as shown in Table 1.

**Table 1:** Information about mentors’ cohorts 2010–2012^a^.

Characteristic	Mentor cohort 2010, no. (% of 16)	Mentor cohort 2011, no. (% of 21)	Mentor cohort 2012, no. (% of 16)
**Gender**			
Female	4 (25.0)	6 (28.6)	5 (31.3)
Male	12 (75.0)	15 (71.4)	11 (68.7)
**Academic rank**			
PhD	6 (37.4)	6 (28.6)	6 (37.4)
Privatdozent	5 (31.3)	6 (28.6)	5 (31.3)
Professor	5 (31.3)	9 (42.8)	5 (31.3)
**Scientific area**			
Basic scientist	6 (37.5)	6 (28.6)	6 (37.5)
Clinical scientist	10 (62.5)	15 (71.4)	10 (62.5)
**Research center**			
Cardiovascular Research Center	6 (37.4)	6 (28.6)	7 (43.8)
Center for Inflammation, Infection and Immunity	1 (6.3)	3 (14.3)	2 (12.5)
Hamburg Center of NeuroScience	3 (18.8)	6 (28.6)	5 (31.2)
University Cancer Center Hamburg	5 (31.2)	4 (19.0)	2 (12.5)
Center for Health Care Research	1 (6.3)	2 (9.4)	0 (0)
**Number of mentees/mentor**, Mean (SD)	2.31 (1.14)	1.76 (0.89)	2.13 (1.03)
**Available mentors, who have not been chosen**, no. (% of total mentors of the respective cohort)	2 (12.5)	1 (4.5)	3 (18.8)

^a^ Information are given for mentors with mentee(s) only.

### Focus groups

We conducted two focus groups that were based on semi-structured interviews with open-ended questions. Participants were asked to reflect their MSD experience and used the following prompts to guide the discussion during focus groups: *“What have been the benefits of the MSD event in your view*?*”*, *“How did you feel regarding the surrounding and atmosphere during MSD*?*”*, *“What was the time frame like in your experience during MSD*?*”*, *“To what extent is MSD able to facilitate a long-term mentoring relationship*?*”*.

We contacted all students (mentoring cohort 2011: n = 37, mentoring cohort 2012: n = 34) and mentors (2011: n = 21; 2012: n = 16) who participated in the previously described MSD by email and invited them to participate in separate focus groups. In total 15 students (5 female) and 15 mentors (2 female) participated in the focus groups. Informed consent was received before the focus groups started. According to recommendations, focus-groups were heterogeneous regarding gender. Each session lasted 60–90 minutes [24] and was led by an experienced moderator. Furthermore one author (JG or GK) attended to take notes using a pre-assembled matrix to delineate the sense of consensus within the focus group as suggested by Onwuegbuzie et al. [25]. The focus groups were audiotaped and transcribed verbatim according to predefined and accepted transcription rules [8, 19]. Qualitative research was performed in accordance with the consolidated criteria for reporting qualitative research (COREQ) of Tong et al. [26].

### Survey

All mentoring cohorts (2010, 2011, and 2012) were asked to complete a set of items with regard to characteristics of their mentoring relationship after six months of participation in the mentoring program for excellent students. To explore the effect of MSD, the three cohorts were assigned to two groups: Cohort 2010 was assigned to the group without MSD, whereas the cohorts 2011 and 2012 were assigned to the MSD-group. Following our applied definition of mentoring and desirable characteristics of mentors that are recommended by Berk et al., two of the authors (JG and ES) developed the items to reflect a comprehensive assessment of the mentoring relationship between students and faculty mentors [1, 5, 6]. Items were written to meet established criteria [27]. The items were rated on a 1–6 Likert scale ranging from “disagree strongly” (= 1) to “agree strongly” (= 6). All authors reviewed the items several times to verify that questions were understandable and clear until consensus was reached. Participation in the survey was voluntary. Students signed an informed consent to participate. No personal identification was required.

### Continuance of mentoring relationship

Furthermore the duration of the mentoring relationship was considered as an outcome measure. To assess the 1-year continuance of the mentoring relationship we sent an email to all mentees after 12 months of mentorship asking whether they would like to continue the mentoring relationship. In case we did not receive a response after two reminder emails we contacted the respective mentor to clarify the current state of their mentoring relationship.

### Data analysis

#### Focus groups

Each focus group was analyzed by using conventional content analyses with inductive categorization. Data analysis started with reading the transcripts repeatedly to gain a sense of the whole. Subsequently three of the authors (CM, GK, and JG) reviewed the data word by word to identify key concepts and generate labels of codes independent from each other. Next, CM, GK and JG sorted codes into categories, which were reviewed by all authors. During this process we developed final definitions for each category and code. Consensus was reached through considering the matrix, which was completed by one of the researchers (CM, GK or JG) during the focus groups [25]. We chose excerpts to exemplify each category and translated them into English. Qualitative data was analyzed using the software MAXQDA 10.

#### Survey

Descriptive statistics were generated to provide an overview. Pearson’s chi-squared tests were used to evaluate for differences in gender between the mentoring cohorts. To compare the answers of students in the two groups (no MSD vs. MS), two independent *t*-tests were carried out for the six items illustrating the mentoring relationship from the mentee’s point of view. To explore the possible effect of gender, a second independent *t*-test with gender as group variable was computed. In addition, a two-factorial multivariate analysis of variance (MANOVA) with MSD and gender as the main factors and MSD and gender as an interaction term was conducted to evaluate the responses of the two groups (no MSD and MSD) to the six items illustrating the mentoring relationship. Gender was also included in the analyses because the mean was different for males and females among some items. We used another chi-squared test to analyze for differences of the 1-year continuance among the group matched via MSD and the no MSD group. For the statistical analyses PASW SPSS 18.0 was used.

### Ethics statement

The study was carried out in accordance with the Declaration of Helsinki. The study protocol was approved by the Dean of the University Medical Center Hamburg-Eppendorf, Hamburg, Germany since the research protocol was not deemed to be biomedical or epidemiological research. One week before the MSD event and the focus-groups participants received a fact sheet of the study and a consent form by email. Participation in the study was voluntary. There was no disadvantage to those who chose not to participate. At the beginning of the MSD event and the focus-groups the study was fully explained by one of the researchers (JG). Only mentees and mentors who gave written informed consent freely were able to participate in the study. Data were anonymized by one author (JG), who had access to identifying participant information. None of the other authors had access to identifying participant information. The Dean of the University Medical Center Hamburg-Eppendorf, Hamburg, reviewed the study and consent procedure. Participants were able to opt-out of the study without experiencing disadvantages until data were anonymized. Quantitative data underlying the study findings are freely available.

## Results

### Focus groups

We conducted four focus groups in total (two with mentees, two with mentors). 15 students (mentoring cohort 2011: n = 8; mentoring cohort 2012: n = 7; n = 5 female) and 15 mentors (n = 2 female) participated in separate focus groups. We identified several themes focused on benefits of MSD, the environment and the time-frame. We found slight differences in the responses by group (mentors vs. mentees). Thus codes and illustrative quotes are presented separately for mentors and mentees.

### Benefits of MSD

We identified numerous benefits of MSD. Most commonly, mentors and mentees mentioned that MSD helps to account for the ‘chemistry’ of the relationship and personal component. One mentor stated:

*With MSD… you easily find out whether the chemistry does fit or not*.

Likewise one mentee said:

*Chemistry is the most important*. *It is not only the area of research and what the mentor is doing in his lab all day*, *but how he is as a person*. *Everybody is different and you cannot get this [information] from the online profile*. *And it is the most important to notice if you get along with each other*.

All mentees and mentors emphasized that MSD is very useful to get a first impression. Participants stated that finding out about the unique personal connection is of great importance for future mentoring relationships:

*The main aspect is that both participants get a personal impression…that is extremely important in my opinion*.

Furthermore MSD helps to match the expectations of both parties involved in the mentoring relationship. Both, mentees and mentors pointed out the importance to compare each other’s expectation with regard to academic interest and the role of the mentor, respectively mentee. One mentor stated:

*I think the main benefit is that both the mentor and the mentee are able to clarify their expectations with regard to the mentoring relationship*. *Within a short time I may present my way of supporting as a mentor and clarify if the expectations match*.

One mentee compared MSD to the mentor profiles and said:

*In comparison with the online profiles [speed mentoring] is more important for the decision*. *The mentor profiles were good to get an overview about what the mentors are doing [area of research and specialization] and then watch their research websites to find out which area of research I am interested in and afterwards meet them in person*.

In addition, mentors mentioned that MSD enables them to avoid ambiguity with regard to the detailed area of research and daily routines. One mentor stated:

*A very important thing is that there are mentors who are focused on experimental biomedical research and some with an emphasis on clinical research*. *In case I conduct clinical research I could send a mentee who would like to work with mouse models for example to a mentor experienced in this research approach*. *With speed mentoring you can avoid ambiguity*.

MSD also benefits from an overview of several candidates. Participants frequently outlined the efficiency and comparability inherent to this matching strategy:

*Within a short period of time you had the chance to meet several scientists*. *You got to know what exactly they are doing and what you are interested in*. *They usually have different projects and speed mentoring is helpful to get an overview*. *It was extremely timesaving and efficient*.

Furthermore MSD serves as helpful decision aid. Participants mentioned the advantage of the bidirectional process in which both parties choose their preferred candidates. Mentees experienced the mentors’ choice of their person as a pleasant feedback in particular when considering the high academic ranks of mentors (professors, directors) and their numerous duties. One mentee said:

*I think it is good*, *that the mentors have to make an ‘active’ choice*, *too*. *Thereby you get the feeling*, *that the mentor wants you as well*, *and you don`t feel like you impose on him*. *This feedback is really helpful*.

Mentees mentioned two additional categories which were not described by the mentors. One mentee said that self-reflection was increased through structured questions of the mentors during MSD. Furthermore some mentees reported that the mentors’ expectations regarding mentorship and the students’ performance became clear.

#### Environment and time-frame of MSD

We identified two key aspects with regard to the environment of MSD: Atmosphere and time-frame. Several mentees perceived the atmosphere as tensed and exciting. A few mentees said it felt similar to a job interview. Additionally the hierarchy gap played a role. Likewise mentors mentioned the ‘interview character’ and obvious hierarchy. Mentors reported an open-minded atmosphere when reflecting on their MSD experiences. Both mentees and mentors emphasized that they adapted quickly to the 5 minute sessions and the mentees’ initial nervousness decreased (Table 2).

**Table 2:** Categories, codes and representative quotes with regard to the environment and time-frame of MSD.

Focus Group	with mentors (*n* = 15)	with mentees (*n* = 15)
Category and codes	Illustrative quotes	Illustrative quotes
**Atmosphere was perceived as positive (mentors) or as tensed and exciting (mentees)**	Perceived as positive: *Considering the atmosphere I think it was positive*, *that students came with great open mindedness*, *asked questions and described their interests and ideas seriously*.	Perceived as tensed and exciting: *The atmosphere was a bit tensed*, *but within a tight time schedule you probably cannot avoid this*. *We are young students facing experienced academic physicians*. *Probably you can’t avoid nervousness on both sides but definitely students deal with it more severely*.
**Atmosphere was similar to “interview situation”**	*Some students were prepared like for a job interview*. *After you got over with that*, *you could start talking about the real things*.	*If you opt for a certain mentor you want to perform well to be chosen by this mentor*. *Thus you try to present yourself as good as possible*.
**Atmosphere was influenced by hierarchy gap**	*I think the personal aspect in the mentee-mentor-relationship is important*. *Mentees are a little bit nervous facing this conversation*. *The hierarchy in medicine is strong and when I asked for their last holiday*, *for example*, *I had the impression*, *they think*, *this doesn`t belong to the subject*.	*You start the conversation with a big distance*, *a student and an experienced professor*. *But that’s the clou*. *It was a helpful decision criterion*: *Did I get to the point of feeling comfortable despite the distance or did the gap remain*?
**Time-frame facilitated focus on essentials**	*The time limit [5 minutes] helps that both focus on the main aspects and I think …this is extremely important*.	*The limited time was really fine*. *It is enough to find out the important things*. *Both parties get to know each other*. *Then you approach the next mentor*.

#### Facilitation of long-term mentoring relationships

When we asked the participants in how far MSD might facilitate long-term relationships mentors identified two major categories (fulfillment of expectations, enhanced commitment). Mentees mentioned fulfillment of expectations only. Participants emphasized that the ‘real mentorship’ evolves after MSD as mentors and mentees get to know each other. Still most participants considered MSD as an important basis to build on a serious, long-term mentoring relationship. One mentor stated:

*I think*, *if mentees get their selected mentor*, *they are even more motivated*. *They feel more comfortable regarding the first meeting with their mentor because they have an idea what to expect*. *And very likely they are glad that they succeeded to get their preferred mentor*.

Both mentees and mentors pointed out that the mentoring relationship and process met their expectation. One mentor reported:

*The matching was ideal*. *All three of my mentees met my expectation in terms of high motivation for scientific work and mentoring for one and half year by now*.

Likewise one mentee said:

*Speed mentoring provides a sound basis for good*, *long-term [mentoring] relationships later on*. *The mentoring relationship with my mentor progressed and works very well*.

In addition mentors hold the opinion that MSD leads to high commitment and results in better team work among the mentees. Finally, mentors gave examples for the success of the mentoring relationships originated in MSD in terms of substantial career moves of their mentees:

*It became clear*, *that these [mentees] participate in a graduate school … or applied successfully for a grant from ‘Studienstiftung des deutschen Volkes’ [largest German institution to sponsor young talents] or ‘Cusanus Werk’ [scholarship body of the Catholic Church]*.

### Survey

The response rates of the survey were 78.4% (n = 29) for mentoring cohort 2011, 94.6% (n = 35) for mentoring cohort 2012 and 85.2% (n = 29) for mentoring cohort 2013. Neither the three mentoring cohorts differed significantly in age (*F*(2, 90) = .273 p = .762) or gender (χ^2^(2, N = 93) = .277, p = .871) nor did the two groups MSD vs. non MSD show statistically significant differences in age (*t*(91) = .340 p = .735) or gender (χ^2^(1, N = 93) = .000, p = .988). Table 3 illustrates gender ratio and age (Mean, SD) of the mentees enrolled in this study.

**Table 3:** Demographic characteristics of the mentees’ cohorts 2010–cohort 2012^a^.

	Mentee cohort 2010, no. (% of 29)	Mentee cohort 2011, no. (% of 35)	Mentee cohort 2012, no. (% of 29)
Matching method	No MSD	MSD	MSD
**Gender**			
Female	15 (51.7)	17 (48.6)	16(55.2)
Male	14 (48.3)	18 (51.4)	13 (44.8)
**Age**			
Mean (SD)	22.52 (2.52)	22.51 (3.40)	22.03 (2.61)

^a^ Information are only given for mentees who completed the survey after 6 month of mentorship.

Table 4 shows the mentees’ mean responses (and 95% confidence intervals, CI) to six items on the quality of their mentoring relationships after six month of mentorship. To explore the effect of MSD, the three cohorts were assigned to two groups: Cohort 2010 was assigned to the group without MSD, whereas the cohorts 2011 and 2012 were assigned to the MSD-group. Mentees who participated in MSD reported higher mean satisfaction scores regarding the mentorship quality than mentees who chose their mentors via online profiles only.

**Table 4:** Differences of mentorship quality from the mentees’ point of view categorized by matching method (no mentoring speed dating (no MSD) vs. mentoring speed dating (MSD)).

	No MSD^a^ (n = 29)	MSD^b^ (n = 64)	MANOVA results for factor matching method		
Items^c^	M [95% CI]	M [95% CI]	*F*	df	p
My mentor was easily **accessible**.	4.78 [4.43–5.13]	5.17 [4.92–5.42]	2.689	1	p = .105
My mentor is **committed** to mentoring.	5.00 [4.66–5.34]	5.50 [5.30–5.70]	5.768.	1	p = .019*
I have **confidence** in my mentor’s professional integrity.	4.90 [4.53–5.27]	5.27 [5.04–5.49]	2.045	1	p = .156
My mentor demonstrated content **expertise** in my area of need.	5.66 [5.44–5.78]	5.75 [5.60–5.90]	.553	1	p = 459
My mentor provided constructive and useful **feedback**.	4.58 [4.03–5.14]	5.16 [4.91–5.40]	4.454	1	p = .038*
Altogether I am **satisfied** with my mentor.	4.97 [4.57–5.37]	5.42 [5.20–5.64]	3.095	1	p = .082

^a^ Mentee cohort 2010 (n = 29)

^b^ Mentees’ cohorts 2011 (n = 35) and 2012 (n = 29) together

^c^ Rated on a 1–6 scale ranging from “disagree strongly” (= 1) to “agree strongly” (= 6).

* p <.05

The independent *t*-test using MSD as the group variable, revealed statistically significant differences for three items: “My mentor is committed to mentoring” (*t*(91) = -2.66, p = .009), “My mentor provided constructive and useful feedback” (*t*(86) = -2.22, p = .029) and “Altogether I am satisfied with my mentor” (*t*(91) = -2.19, p = .031). The result of an independent *t*-test using gender as the group variable revealed that male students scored higher than female students in all six items. A statistically significant difference was found for one item only: “Altogether I am satisfied with my mentor” (*t*(91) = -2.10, p = .039). At a Bonferroni corrected p = .008 no significant difference could be found any longer.

In order to get a complete picture of the factors MSD and gender on mentoring relationship, a MANOVA was conducted. The MANOVA showed no statistically significant effect, neither of the factors (MSD (*F*(6, 79) = 1.701, p = .132) and gender (*F*(6,79) = 1.622, p = .152) nor the interaction MSD*gender (*F*(6, 79) = 1.604. p = .157). On item-level, the items “My mentor is committed to mentoring” (*F*(1) = 5.768. p = .019) and “My mentor provided constructive and useful feedback” (*F*(1) = 4.454. p = .038) showed a statistically significant effect of the matching method (no MSD vs. MSD; Table 4).

**Fig 1:**
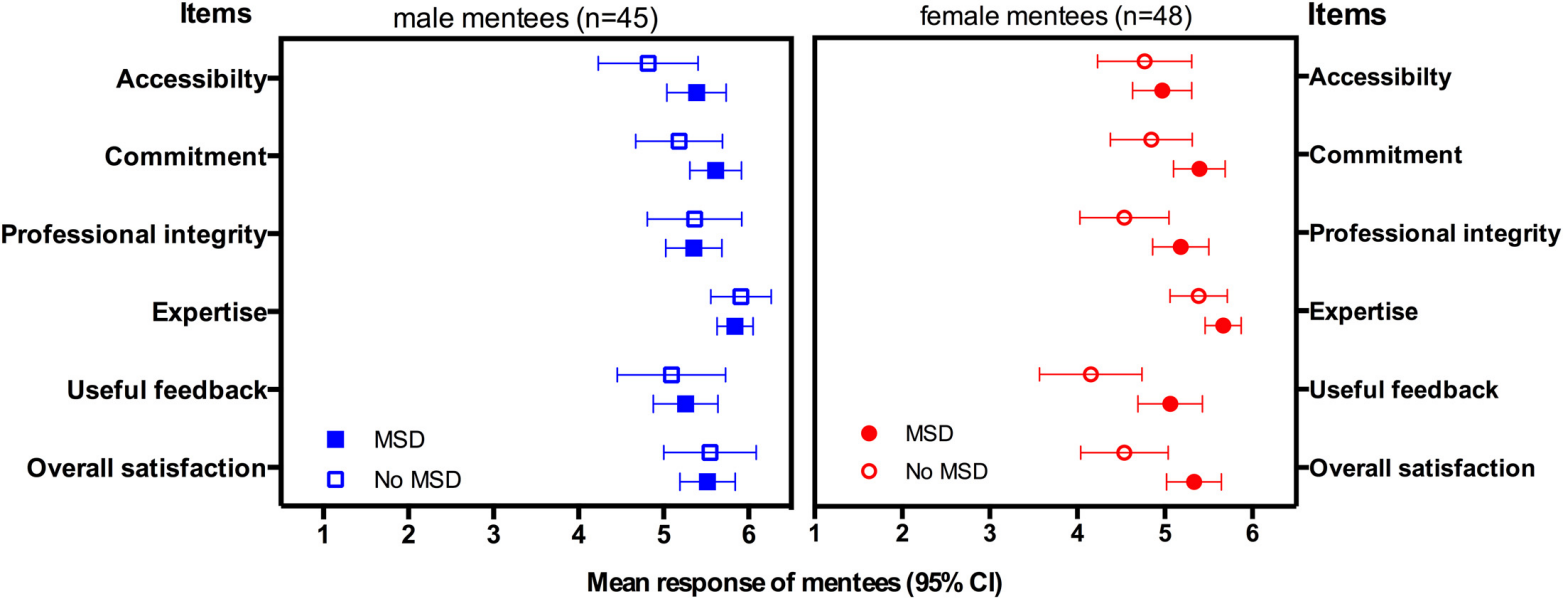
Mean responses of mentees to items on mentorship quality. All items were rated on a 1–6 scale ranging from “disagree strongly” (= 1) to “agree strongly” (= 6). Means and 95% confidence intervals (95% CI) of the six items are given for gender and matching method. MSD = mentoring speed dating; no MSD = no mentoring speed dating.

Gender as a factor showed a statistically significant effect among the items “I have confidence in my mentor’s professional integrity” (*F*(1) = 5.061. p = .027), “My mentor demonstrated content expertise in my area of need” (*F*(1) = 5.993. p = .016), “My mentor provided constructive and useful feedback” (*F*(1) = 4.970. p = .028), and “Altogether I am satisfied with my mentor” (*F*(1) = 7.476. p = .008). Therefore mean responses are displayed separately for gender (female: n = 48, male: n = 45) as well as matching method (MSD: n = 64; no MSD: n = 29) in Fig 1.

Overall, the ratings of female mentees who were matched via MSD were higher in comparison to female mentees who chose their mentors via online profiles (no MSD). In contrast, male students who participated in MSD rated only two items higher in comparison to the “no MSD group”. The interaction of matching method and gender showed no significant effect on item-level.

### Continuance of mentoring relationship

**Fig 2:**
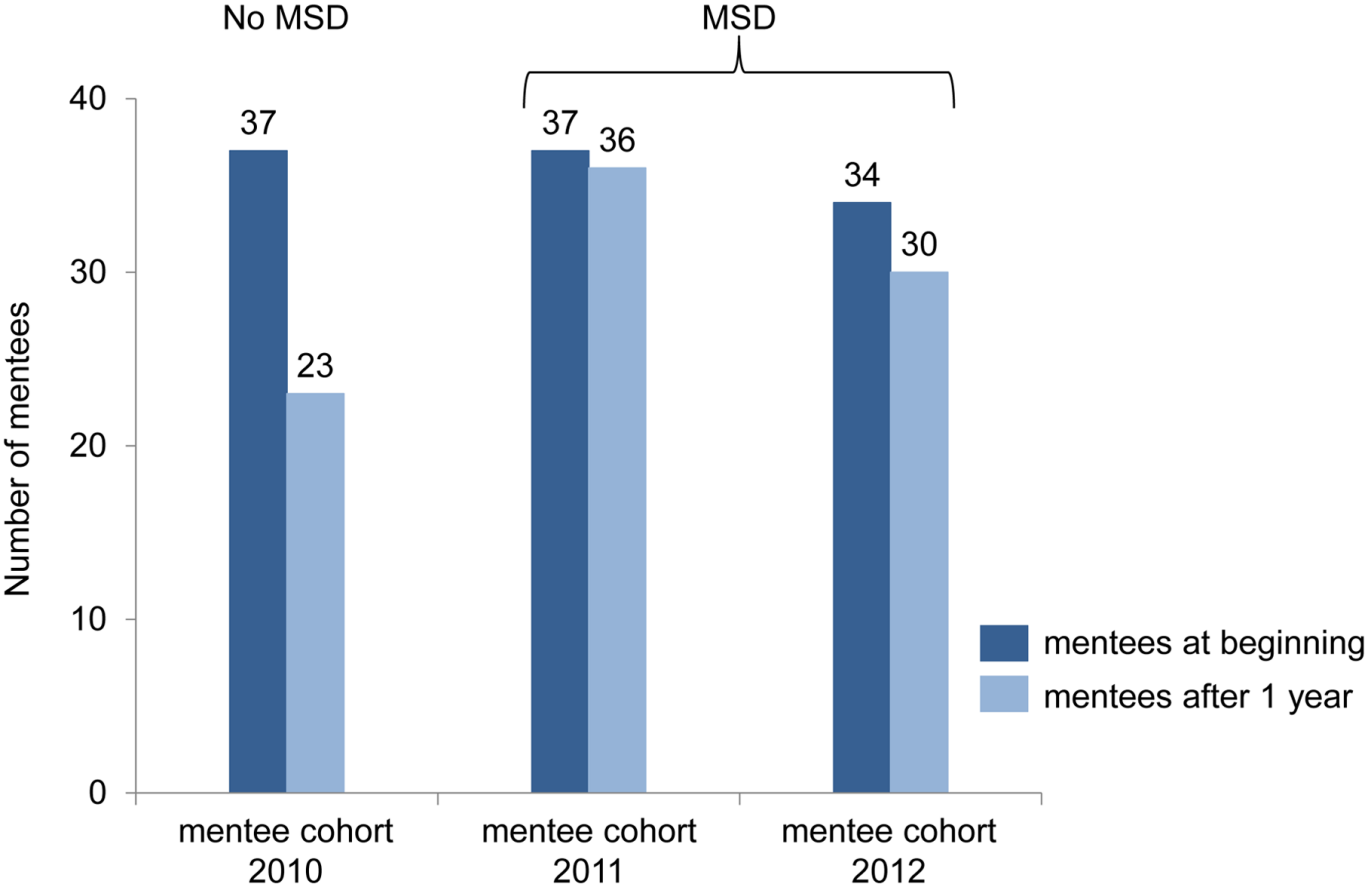
Frequencies of mentees’ dropout after 1 year mentorship. Cohort 2010 constituted the “no mentoring speed dating (no MSD)” group and the cohorts 2011 and 2012 constituted the “mentoring speed dating (MSD)” group.

We analyzed the 1-year continuance of the mentoring relationship as a criterion for the success or failure of mentorship. The percentage of mentees who quitted mentoring after one year differed significantly by matching method (MSD vs. no MSD; χ^2^(1, N = 108) = 15.912, p < .000). More than one third of the mentees who chose their mentor via online profiles of the mentors quitted mentoring after one year. In comparison only one mentee of each mentoring cohort matched via MSD resigned the mentoring relationship with his or her mentor after one year (Fig 2). Three mentees of cohort 2012 were excluded from the program because they did not attend a compulsory event of the program due to private reasons.

## Discussion

Previous research has shown that successful mentoring is based on matching the mentee and mentor according to personal and attitudinal similarities [13, 15, 28–30]. A qualitative study by Straus et al. revealed that having the option of choosing the mentor would increase the mutual trust and decrease the possibility of failure [20]. Furthermore, being able to contribute to the matching process is associated with greater mentorship quality and role modeling [13, 31, 32]. In summary, the unique personal connection or ‘chemistry’ between mentor and mentee is a crucial factor for the success and longevity of the relationship [29]. Although several studies have addressed the importance of mentee-mentor compatibility in terms of personal, professional and attitudinal similarities for effective mentoring [19, 20, 29, 33], less detail has been available on the initiation of mentoring relationships [19, 20]. Finding a suitable mentor can be difficult and poses a challenge to mentees’ perseverance [29].

Formal mentoring programs in academic medicine usually employ three major approaches to match mentors and mentees: randomly assignment of mentees to mentors by an administrator, selectively matching based on certain criteria such as clinical specialty, area of research, personal attributes and/or other aspects conducted by an administrator, or mentees are allowed to choose their mentors on the basis of previously mentioned criteria [1, 18]. Recently speed-mentoring has been recommended as an innovative approach to initiate mentoring relationships [21]. To our best knowledge MSD that integrates with formal mentoring programs in academic medicine has not been explored. The presented MSD approach addresses the important need of choosing the right mentor-mentee matches so that elements of trust and commitment are enhanced in the relationship. Our study fills this gap and explored mentees’ and mentors’ experience with MSD and investigated the impact of MSD on the perceived mentorship quality in a formal mentoring program. Furthermore it takes into account the 1-year continuance of mentorship associated with two different matching procedures. Our study provides information for institutions who intend to introduce MSD to facilitate long-term relationships as well as guidance for future participants of MSD events.

A recent qualitative study across two academic health centers described that failed mentoring relationships were characterized by several aspects including poor communication, lack of commitment to the mentoring relationship and lack of personal similarities [33]. Our quantitative data are in line with these results: students who chose their mentors via MSD rated their mentors’ commitment and their feedback significantly higher than mentees who found their mentors via online profiles. Furthermore their overall satisfaction with their mentor was higher. We found no overall difference with regard to the ratings of the mentors’ accessibility, his expertise and professional integrity. Interestingly, when categorized by gender and matching procedure female students in the MSD group reported higher satisfaction with all six mentorship aspects in comparison to female students who chose their mentors via online profiles (Fig 1). By contrast, male students rated only the commitment of the mentor, his accessibility and the mentor’s feedback higher than their peers in the “no MSD group”. One reason for this effect might be that the absolute satisfaction of male students without MSD was already very high. Except the item regarding accessibility of the mentor all items were rated by male students in the no MSD group > 5 (with 6 as highest possible value).

In our study participants reported that MSD largely benefits from the bidirectional process in which both parties chose their preferred candidates. Thus the MSD process allows mentees and mentors to have more influence on the matching process. Two US studies that explored formal mentoring programs in organizational settings found that perceived input into the match was associated with greater mentorship quality, greater career mentoring [13] and greater mentorship satisfaction by the mentees [34]. These results correspond with our findings that mentees who attended MSD reported increased satisfaction with their mentors as compared to students who chose their mentors via online profiles (Table 4).

The continuance of mentorship serves as an apparent indicator for the success of mentorship. The differential mentoring program offered at the UKE is designed to accompany students until they finish medical school, still both parties are able to quit the mentoring relationship at the end of each semester [4]. Comparing the 1-year continuance of the mentoring relationships of all mentoring cohorts we found a striking difference (Fig 2). Among mentees who chose their mentor based on online profiles (no MSD group) 14 (38%) out of 37 mentoring relationships were terminated in contrast to 2 (3%) out of 68 mentoring relationships based on mentoring speed dating (MSD group). Interestingly Cook et al. reported that speed-mentoring did not seem to stimulate ongoing mentoring relationships [21]. In their study seven junior faculty members (mentees) and six senior faculty mentors participated in the speed-mentoring event. Both mentees and mentors reported that time was well spent, but only two mentees contacted a participating mentor afterwards. Cook et al.’s different findings may be explained by the absence of a formal mentoring program.

A recent qualitative study across two medical schools identified lack of commitment and communication as characteristics of failed mentoring relationships [33]. Considering our quantitative data the significantly higher ratings for the mentor’s commitment and his feedback among the MSD cohort (Table 4) may explain the findings of far less failed mentoring relationships that were initiated via MSD (Fig 2).

A recent systematic review of the mentoring literature described the complexity of the personal connection for mentoring relationships. The authors reported that mentoring relationships were potentially enhanced by similar interests and ideals [19]. Our qualitative data provided unique details on the perceived importance of the “right chemistry” and the great benefit of MSD to find out about the academic and personal interests of each other. Several participants of our focus-groups reported that expectations from both sides could be made clear. In their point of view MSD was a valuable decision-aid and the input into the match by the mentor was highly appreciated by the mentees as a welcome indicator for the mentor’s commitment. According to mentees and mentors in our study MSD largely benefits from the efficiency and comparability between candidates and the time frame helps to focus on the important aspects.

Mentoring in academic medicine is challenged by clinical, research and administrative demands [9, 10]. Thus effective mentors are a precious resource. Therefore we were particularly interested in the perspectives of both (mentee and mentor) concerning the subsequent long-term mentoring relationships that are initiated via speed-mentoring. Of particular interest was that nearly all participants of the focus-groups were convinced that MSD provides an excellent starting point for long-term mentorship to build on.

Our study has several limitations. First focus groups are not suitable for generalization. Even though group interaction distributes substance to the topic, individual opinions can occur. Second a selection bias is possible: we only interviewed participants of one medical school. Third the students were high-performing and invited exclusively to the Mentoring Program for Excellent Students [4]. Thus the possibility of social desirability is given. Another limitation concerns a possible bias in the fact when introducing the MSD into the well-established program of mentoring for excellent students, that other measures interfered, which may have influenced the results. To avoid confounding results no other component of the mentoring program was changed: (i) The mentors and mentees originated from the same population, the ratio basic to clinical scientists (mentors) and female to male (mentees and mentors) as well as the mean age (mentees), the academic rank (mentors) and the mean number of mentees/mentor were approximately the same for both groups (MSD vs. no MSD; Tables 1 and 3), (ii) the instructions for mentors and mentees were kept rigorous the same, and (iii) other program components such as annual retreat did not change. Still we believe that limitations mentioned above were partly overcome by using multiple data sources [35]. Reliability and validity can be established by peer-debriefing, longitudinal observation and triangulation [36]. The approach of conventional content analysis can be used when existing theories or literature is limited. The information emerges directly from the participants without predefined categories [36–38]. Furthermore, conventional content analysis allows for a natural and content related depiction of the focus group interaction and reduces the risk of research bias [38].

## Conclusion

The present data suggest that MSD is a valuable matching tool for a medium group size. It helps to overcome common mentoring barriers such as lack of fit between mentor and mentees [39] and vulnerability of mentees for example that the mentee feels rejected when the mentor cancels meetings [40]. MSD meets recommended strategies to improve mentoring [19] and appears to foster long-term mentoring-relations based on clear expectations and personal connection [33]. The predefined time-frame of 5 minutes was appreciated by mentees and mentors. We recommend MSD as very efficient to initiate effective mentoring relationships. For future studies on matching procedures of formal mentoring programs we suggest to involve more participants of formal mentoring programs at different medical schools.
